# Knowledge and Testing of Hepatitis B Virus Infection and Vaccination Awareness among University Students in Kumasi, Ghana: A Cross-Sectional Study

**DOI:** 10.1155/2024/4052837

**Published:** 2024-05-20

**Authors:** Elizabeth Abban, Emmanuel Owusu, Benedicta Kwakye-Abebrese, Bright Koduah, Hannah Boateng, Emmanuel Ekow Korsah, Alfred Effah, Solomon Akpobi, Emmanuel Avoh-Ackah, Ebenezer Senu

**Affiliations:** ^1^Department of Medical Laboratory Technology, Garden City University College, Kumasi, Ghana; ^2^Department of Medical Diagnostics, Faculty of Allied Health Sciences, Kwame Nkrumah University of Science and Technology, Kumasi, Ghana; ^3^Department of Molecular Medicine, School of Medicine and Dentistry, Kwame Nkrumah University of Science and Technology, Kumasi, Ghana; ^4^Laboratory Department, School of Public Health, KNUST-IVI Collaboration Center, Kwame Nkrumah University of Science and Technology, Kumasi, Ashanti Region, Ghana; ^5^Department of Clinical Chemistry, Empire City Laboratories, Brooklyn, New York, USA

## Abstract

**Background:**

Hepatitis B is a potentially life-threatening liver infection caused by the hepatitis B virus (HBV) and major global health problem, affecting millions of people globally. Whilst college or university students could serve as a positive vehicle that may aid in the propagation of education in the communities, there is currently paucity of data on knowledge of HBV and vaccination awareness among university students in Ghana. This study therefore assessed knowledge on hepatitis B virus infection, testing, and vaccination awareness among science and non-science students in Ghana.

**Method:**

This cross-sectional study included 386 students from the Garden City University College, Kumasi, Ghana, from February to June 2022. A well-structured questionnaire was used to obtain data on knowledge, testing, HBV vaccination status, and sociodemographic characteristics of participants. All statistical analyses were done using SPSS Version 26.0 and GraphPad prism version 8.0. A Chi-square test statistic and logistics regression analyses were used to determine factors associated with study variables among study participants. *p* value of <0.05 and 95% confidence interval were considered statistically significant.

**Results:**

About half (51.5%) of the participants had adequate knowledge on hepatitis B infection with 22.3% demonstrating poor knowledge on hepatitis B infection. A little more than average (51.6%) previously tested for hepatitis B (HBV) whilst 32.9% were highly aware of hepatitis B vaccination and 33.9% were not aware of hepatitis B vaccination. This study found that ethnicity of students (*p*=0.0020), family history of hepatitis B infection (*p*=0.0160), and academic cumulative weighted average (CWA) (*p*=0.0020) were significantly associated with knowledge about hepatitis B infection. Also, students reading science-related programs had more than twice the odds (aOR = 2.56, 95% CI (1.03–5.08), *p*=0.0210) of having tested for HBV infections before compared to students who read non-science programs. Furthermore, sex (*p* < 0.0001), family history of HBV vaccination (*p*=0.0260), CWA (*p*=0.0060), and the program of students (*p*=0.0020) were significantly associated with awareness of HBV vaccination.

**Conclusion:**

Knowledge of HBV infection among university students is satisfactory but awareness of HBV vaccination and testing is poor. There is a need to enhance educational interventions to improve the general knowledge of HBV infection, testing, and vaccination in Ghana especially among non-science students.

## 1. Introduction

Viral hepatitis B infection is a serious health condition that poses a significant public health concern globally, especially in developing countries [1]. Hepatitis B is described as a liver infection caused by hepatitis B virus (HBV), a member of the *Hepadnaviridae* family of viruses that can be transmitted through blood, blood products, semen, and other body fluids, as well as from mother to newborn during childbirth [2–4]. Hepatitis B infection can lead to chronic and potentially life-threatening conditions including hepatomegaly, cirrhosis, and hepatocellular carcinoma and also death [1].

HBV infection is thought to affect one-third of the world's population, with an estimated 290 million chronic carriers worldwide in 2010 [5]. Moreover, over 1.34 million deaths were reportedly attributed to viral hepatitis alone in 2015, and this was mostly caused by cirrhosis, which was directly responsible for 720,000 deaths, and hepatocellular cancer [1]. In 2019, estimated 296 million people worldwide were living with hepatitis B [6].

The prevalence of chronic hepatitis B infection is highest in the Western Pacific region and sub-Saharan region, where 116 million people and 81 million people, respectively, are chronically infected [1]. Thus, Western Pacific region and sub-Saharan region account for more than 75% of the worlds chronic HBsAg carries [1, 7]. The high prevalence of HBV in Africa, including Ghana, can be attributed to poverty and a lack of understanding about the disease [8]. Previous study by Agyemang and colleagues shows 12.3% prevalence rate of the HBV infection, as detected by HBsAg seropositivity making it one of the highest globally [9]. An unsettling pattern can be seen in the analysis of the HBV prevalence/incidence data from the Ghana Health Service (GHS) district health information management systems (DHIMS2). Suspected cases, confirmed cases, and deaths from HBV complications were the three categories for reported HBV infection in the DHIMS database. The trend indicates rising incidence and prevalence across the nation. There were 3508, 4419, and 7324 confirmed cases at the national level in the years 2012, 2013, and 2014, respectively [10]. For the three years, there were 164, 101, and 131 reported HBV-related deaths, respectively [10].

Currently, there is no specific antiviral therapy recommended for people with acute hepatitis B disease as over 95% of infected immunocompetent adults recover spontaneously. However, the Food and Drug Administration has approved interferon-*α* (standard and pegylated) and oral antiviral agents (entecavir, tenofovirdisoproxil fumarate, tenofovir alafenamide, and non-preferred lamivudine, adefovir dipivoxil, and telbivudine) for the treatment of chronic hepatitis B. The current treatments for chronic hepatitis B can enhance long-term survival and quality of life, slow or prevent the progression of cirrhosis, and lower the incidence of liver cancer; however, they are not curative. Therefore, the majority of those who begin hepatitis B treatment must do so forever. The challenges and complexity of patient treatment are heightened by the side effects of the medications and the necessity for continuous observation. Consequently, the best strategy for preventing hepatitis B is vaccination. Given the cost-effectiveness and benefit-cost ratios of vaccination to alternative interventions, hepatitis B vaccines are the most efficient, safe, and cost-effective means of preventing and controlling the disease [11, 12]. Hepatitis B vaccine coverage of three doses reached 85% worldwide in 2019, compared to around 30% in 2000 [13]. The effective implementation of hepatitis B vaccination programs will result in a significant decline in the rate of HBV carriers as well as morbidity and death associated with hepatitis B.

On the contrary, due mostly to financial constraints and illiteracy, HBV unawareness, access to screening, and immunization, hepatitis testing and vaccination remained low in resource-limited nations like Ghana.

Although Ghana has made a progress in lowering disease-related mortality and morbidity by combining the hepatitis B vaccination with the diphtheria, tetanus, pertussis (DPT), and *Haemophilus influenzae* type b (Hib) vaccines to create a pentavalent vaccine, majority of the population remain uncovered [14]. There is still issue of high cost of vaccine and lack of awareness of hepatitis B testing and vaccination [15]. Previous studies examined the level of HBV knowledge and vaccination status among medical professionals and science students [14, 16, 17], neglecting the views of the non-medical and non-science student population. This could hinder efforts in promoting hepatitis B vaccination among Ghanaian university students. This study therefore assessed the knowledge on hepatitis B infection, testing, and vaccination status among both science and non-science university students in Kumasi, Ghana, so as to provide targeted suggestions for future prevention and control of HBV among the population.

## 2. Materials and Methods

### 2.1. Study Design and Site

This study adopted an observational cross-sectional study design. The study was carried at the Garden City University College (GCUC) in Ghana. GCUC is located at Kenyasi in the Kwabre East District in the Ashanti Region of Ghana. GCUC was established in 2001 as college of Information Technology and Management Systems (CITMAS). In January 2004, the Board of Directors voted to convert the college into University College, and it was officially accredited under the name Garden City University College, Kenyase, Kumasi, by the National Accreditation Board in July 2005 to run degree and diploma programs. GCUC currently has 11 departments offering undergraduate programmes in business, health, and applied sciences with a total enrollment of about 1917.

### 2.2. Study Population

The study included regular stream students from both science and non-science programs of the Garden City University College who consented to the study. However, students who were ill or incapacitated to respond to the questionnaire and students who at the time of the study were not within the school premises were excluded from the study.

### 2.3. Sample Size Estimation

The sample size was obtained using the Yamane formula: *n*=*N*/1+*N* (*e*^2^), where *n* is the minimum sample size, N is the population of students at the time of the study (1917), and *e* is the margin of error = 0.05.

(1) $$\begin{array}{c} n=\frac{1917}{1+1917\;\left(0.05^{2}\right)}=331. \end{array}$$

Hence, a minimum of three hundred and thirty-one (331) participants were required for the study. To increase statistical power, three hundred and eighty-six (386) students were recruited for the study.

### 2.4. Ethical Consideration

Before the commencement of the study, ethical approval was sought from the Committee on Human Research, Publication and Ethics, School of Medical Sciences, Kwame Nkrumah University of Science and Technology (CHRPE/SMS/KNUST/CHRPE/AP/369/22). Permission was also sought from the Garden City University College administration. Informed consent was sought from each participant before the commencement of the study. Confidentiality and protection of data were also ensured by making sure collected data were entered with assigned codes but not participants' names and data were also security protected by password.

### 2.5. Variables and Definitions

#### 2.5.1. Dependent Variables

In this study, the researchers examined several dependent variables related to students' understanding of HBV infection. These variables included the students' level of knowledge on HBV infection, categorized as poor, moderate, or adequate. Additionally, the study explored the students' HBV testing status, distinguishing between those who had been tested and those who had not. Furthermore, the researchers assessed the students' awareness of HBV vaccination, categorizing it as low, moderate, or high.

#### 2.5.2. Independent Variables

This study assessed independent variables such as age of participants in years defined as 0-infinity, sex defined as male and female, and religion defined as Christian, Muslim, and Traditionalist. Moreover, independent variables such as marital status defined as single, married, and divorced, cumulative weighted average (CWA) defined as 0–100, ethnicity defined as Akan, Ewe, and Ga-Adangbe. In addition, independent variables including residence defined as rural and urban as well as occupation defined as unemployed and employed were assessed.

#### 2.5.3. Data Collection Instrument and Validation

A well-structured questionnaire with close-ended questions was used to collect data from study participants. The questionnaire consisted sections such as sociodemographic, knowledge on hepatitis B infection, HBV testing, and awareness of HBV vaccination. The questionnaire designed was based on validated questions from previous studies [18–20]. The questionnaire was also pretested in different student population at Kwame Nkrumah University of Science and Technology (KNUST), and all misleading and ambiguity questions were corrected before using it among students at GCUC.

#### 2.5.4. Sampling Technique and Data Collection

A convenience sampling technique was used to recruit study participants. A convenience sampling technique help located the participants who met the inclusion criteria to be recruited in the study. The study participants were first informed of the study objectives, and assurance of anonymity was made. Data collection was done by the research team without any secondary data collection personnel. Thus, no additional personnel were trained for data collection since all data collections were done by the research team. This also limited errors which may rise in explaining the questionnaire to study participants. All data collected were further entered anonymously and sent to the data analyst who was also part of the research team.

#### 2.5.5. Scoring and Grading of Knowledge on Hepatitis B Infection and Awareness of Vaccination

To assess the level of knowledge on hepatitis B infection among study participants, a 15-item structured questionnaire was used. All questions were based on validated questions from previous literature. Participants were given scores based on their response to the questions. A positive response attracted a score of 1 and a negative response attracted a score of 0. The maximum score for knowledge on hepatitis B infection was 15. Since there is no accepted cutoff for grading knowledge on hepatitis B infection, the participants were classified as having poor knowledge if they had a score of 0–9 (≤60.0%) and moderate knowledge if they had a score of 10–12 (61.0–80.0%), and participants that had scores of more than 12 (>80.0%) were classified as having adequate knowledge on hepatitis B infection.

Moreover, to assess the level of awareness of hepatitis B vaccination among study participants, a 10-item structured questionnaire was used. Participants were given scores based on their response to the questions. A positive response attracted a score of 1, and a negative response attracted a score of 0. The maximum score for level of awareness of hepatitis B vaccination was 10. The participants were classified as having low hepatitis B vaccination awareness if they had a score of 0–6 (≤60.0%), moderate hepatitis B vaccination awareness if they had a score of 7-8 (70.0–80.0%), and high hepatitis B vaccination awareness if they had a score of 9-10 (>80.0%).

### 2.6. Statistical Analysis

Collected data were entered, cleaned, and coded using Microsoft Excel 2019. All statistical analyses were done using the Statistical Package for Social Sciences (SPSS) Version 26.0 (Chicago IL, USA) and GraphPad prism version 8.0 (GraphPad software, San Diego, California, USA, https://www.graphpad.com). Categorical variables were presented as frequency and percentages. Descriptive statistics were used to describe the study population. Proportion of knowledge on hepatitis B infection, hepatitis B testing, and awareness of hepatitis B vaccination among study participants was presented by bar charts. A Chi-square test statistic was used to determine factors associated with knowledge on hepatitis B infection, hepatitis B testing, and factors associated with awareness of hepatitis B vaccination among study participants. *p* value of <0.05 and 95% confidence interval were considered statistically significant.

## 3. Results

### 3.1. Sociodemographic Characteristics of Study Participants

Most of the study participants were aged 20–29 years (80.1%) whilst only 6.7% were older than 29 years. Majority were females (61.9%), whereas 38.1% were males. Moreover, majority of the study participants were Christians (81.6%) with few being Muslims (17.4%). Also, majority were single (88.6%). Most of the study participants had CWA less than 70 (78.5%) and majority read science-related courses (82.9%) whilst 17.1% read business-related courses. Majority resided in the urban areas (70.7%) and were unemployed (91.5%) (Table 1).

### 3.2. Knowledge on Hepatitis B Infection among Students

**Figure 1:**
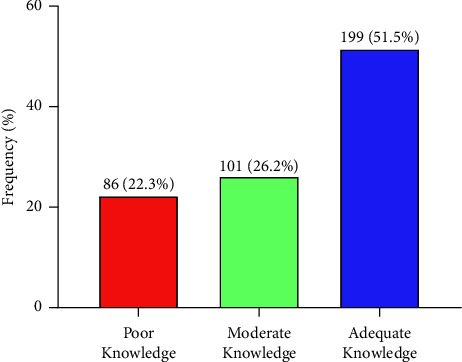
Knowledge on hepatitis B virus (HBV) infection among students.

This study found that little above average (51.5%) had adequate knowledge on hepatitis B infection. About one-quarter (26.2%) had moderate knowledge and 22.3% had poor knowledge on hepatitis B infection (Figure 1).

### 3.3. Proportion of HBV Testing among Students

**Figure 2:**
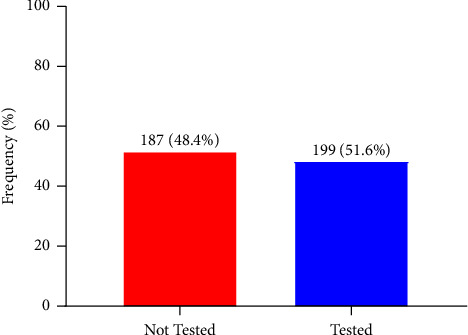
Proportion of HBV testing among students.

This study observed that slightly more than half (51.6%) had tested for hepatitis B (HBV) before whilst 48.4% had never tested for hepatitis B infection (Figure 2).

### 3.4. Awareness of Hepatitis B Vaccination among Students

**Figure 3:**
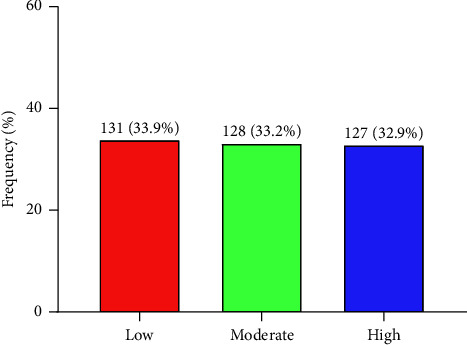
Awareness of HBV vaccination among the students.

Figure 3 depicts the awareness of hepatitis B (HBV) vaccination among university students. Approximately, one-third of 386 study participants were highly (32.9%) and moderately (33.2%) aware of hepatitis B vaccination. Also, one-third of study participants were not aware (33.9%) of hepatitis B vaccination (Figure 3).

### 3.5. Sociodemographic Factors Associated with Knowledge on HBV Infection among University Students

Most participants who had poor and moderate knowledge level on hepatitis B infections were Akans (79.07% and 71.29%, respectively). Among participants who had adequate knowledge, Akans had proportion of 57.29% whilst Ewes had proportion of 32.66%. This study found that ethnicity of students was significantly associated with knowledge about hepatitis B infection (*p*=0.0020). Moreover, a significant proportion of participants who had family history of hepatitis B infection had moderate (15.84%) and adequate (14.57%) knowledge on hepatitis B infection. Participants who had family history of hepatitis B infection were therefore significantly associated with the knowledge of HBV infection (*p*=0.0160). In addition, 34.88% of participants with more than or equal 70 CWA had poor knowledge on hepatitis B infection compared to 65.12% who had CWA of less than 70 with poor knowledge level, whereas 18.59% who had CWA of more than or equal to 70 had adequate knowledge compared to 81.41 who had CWA of less than 70 with adequate knowledge on hepatitis B infection. Academic cumulative weighted average (CWA) range was significantly associated with knowledge about HBV infection (*p*=0.002) (Table 2).

### 3.6. Association between HBV Testing, Sex, and Program of the Study Participants

Majority (90.45%) of the participants who had tested for hepatitis B infection before were reading science programs compared to 9.55% who were reading business programs. There was a significant association between program of study among study participants and hepatitis B testing (*p* < 0.0001). In a multivariate logistic regression model, students reading science-related programs (aOR = 2.56, 95% CI (1.03–5.08), *p* = 0.0210) had over 2-odd chances of having tested for HBV infections.

Of participants who had tested for hepatitis B infection before, most were females (66.33%). Similarly, among those who have not tested for hepatitis B infection before, most were females (57.22%). No significant association was therefore observed between sex and HBV testing (*p*=0.065) among study participants (*p*=0.0654) (Table 3).

### 3.7. Association between Awareness of HBV Vaccination and Sociodemographic Characteristics of Study Participants

Majority of the male participants (54.69%) had moderate awareness of HBV vaccination whilst a greater percentage of females had high awareness of HBV vaccination (76.38%). A significant association was therefore found between sex and awareness of HBV vaccination (*p* < 0.0001). There was also a significant associated between having family history of HBV vaccination and awareness of vaccination (*p*=0.0260). Moreover, the range of cumulated weighted average (CWA) of students was significantly associated with knowledge of HBV vaccination (*p*=0.0060). In addition, the program study of students was significantly associated with knowledge on HBV vaccination (*p*=0.0020). All other sociodemographic variables were not significantly associated with awareness of HBV vaccination (Table 4).

## 4. Discussion

In this study, we examined the knowledge regarding HBV infection among undergraduate students of Garden City University College in Kumasi, Ghana. We also studied the students' HBV testing history and awareness of HBV vaccination. This study found that half (51.5%) of the students had adequate knowledge on hepatitis B infection and about half (51.6%) had tested for hepatitis B (HBV) infection before whilst only one-third of the study participants (32.9%) were highly aware of hepatitis B vaccination. Sociodemographic factors such as ethnicity of students, having family history of hepatitis B infection, and academic cumulative weighted average (CWA) range of students were significantly associated with knowledge about hepatitis B infection. Moreover, students reading science-related programs had over 2-odd chances of having tested for HBV infections. Also, sex, having family history of HBV vaccination, cumulated weighted average (CWA) of students, and the program of study of students were significantly associated with awareness of HBV vaccination.

This study found that near majority of students had adequate or high knowledge of HBV infection (51.5%). Though the knowledge level found in the present study is satisfactory, it is lower than 83.3% reported by Giri et al. from India among medical interns and also slightly lower than 59.5% reported by Aniaku et al. on knowledge of HBV infection among nursing training students in Ghana [14, 21]. However, the knowledge level in this study is higher than 14% reported by Othman et al. in Iraq [22]. These discrepancies may be due to ethnic differences of study participants, sample size variation, and differences in the age groups of subjects recruited for the study. Additionally, the variations observed could be explained by influence of cultural differences on knowledge about infectious diseases such as HBV infection on the backdrop of the fact that culture-specific barriers to health literacy have been reported to contribute to individuals' health behavior in accessing care related to hepatitis [23]. These findings call for the need to adopt strategies for developing culturally tailored resources and programs to enhance knowledge on hepatitis B infection in Ghana. Intensification of educational programs in the Ghanaian universities especially non-science students can contribute high knowledge of HBV infection and improve its preventive measures.

In this study, the low HBV vaccination awareness (32.3%) and knowledge (33.9%) recorded are lower than 44.2% and 73.9%, respectively, recorded by Osei et al. among undergraduate public health students and 37% and 50.8%, respectively, reported by Shrestha et al. among medical college students in Kathmandu, Nepal [17, 24]. This outcome may be very disturbing as inadequate educational programs or low public awareness in our universities on the consequences of HBV may contribute to this finding. Probably it is believed that students at the university do not need to be made aware or educated on such issues as it is already in courses to be learnt in school. This may be true for students pursuing medical and health programs, but non-health students may be lacking in this respect. Moreover, previous research has still shown an underwhelming lack of awareness of vaccination among medical and health students in Nepal [17, 24], hence the need to increase awareness in the universities.

A satisfactory response to the hepatitis epidemic must include HBV testing and diagnosis because it contributes to both treatment and prevention. WHO suggests that in areas with HBV surface antigen of seroprevalence of 2% or 5%, HBV testing should be regularly available and provided to everyone [25]. Additionally, testing offers a chance to connect people in order to prevent transmission through counseling on risky behavior [18]. However, HBV testing rate among students in this study was not satisfactory (51.6%). This result is akin to 53% HBV testing rate found among the USA population [26] and 49.6% HBV testing rate among Ghanaian undergraduate public health students [17] but less than findings observed among hospital staff (81%–70%) in Nigeria [27]. This is concerning because knowing one's status is a requirement for receiving the HBV vaccination. In Ghana, hepatitis B testing and immunization are not paid for by the National Health Insurance program, and consequently payment is required before testing. Additionally, testing for HBV is mostly advised in hospitals for blood donors and patients who are thought to be reactive to hepatitis B [9]. Effective free screening program among university students is a way of reducing transmission of the virus and encouraging vaccination.

This study also analyzed the association between sociodemographic characteristics of study participants, knowledge of HBV infection, and testing and awareness of HBV vaccination. Family history of HBV infection was significantly associated with knowledge of HBV infection and vaccination. Majority of students who had a family history of HBV infection had adequate knowledge of HBV and vaccination awareness. This is consistent with a study by Hwang et al. who similarly found individuals who had a family member with HBV or liver disease had significantly higher levels of HBV knowledge [28]. The CWA range of students was likewise associated with the knowledge of HBV infection and vaccination. Academic brilliance of subjects may influence subject's knowledge of HBV infection and vaccination awareness. Ethnicity was also shown to be associated with knowledge of HBV infection. Larger studies might be required to validate this finding.

Moreover, a student's program of study was significantly associated with HBV testing and vaccination. A greater proportion of students pursing business-related programs had never been tested for HBV nor vaccinated. Issues of public health are less studied in non-science related programs in Ghana. This could be a driving force for ignorance on issues relating to HBV infection, testing, and the subsequent lack of vaccination in these groups. Inclusion of courses in public health and seminars in these programs may help encourage testing among these groups.

This study is limited by the inability to performed robust statistical analyses on some potential predictable variables due to the smaller sample sized employed in the study. Further multicenter studies are needed to establish more significant associations on knowledge, testing, and awareness of hepatitis B vaccination among college or university students in Ghana. This study however contributes significant findings for the further studies.

## 5. Conclusion

Knowledge of HBV infection among university students is satisfactory but awareness of HBV vaccination and testing is poor. There is a need to enhance educational interventions to improve the general knowledge of HBV infection, testing, and vaccination in Ghana especially among non-science students. Developing and implementing national HBV screening and vaccination programs are essential in successfully reducing morbidity and mortality caused by HBV infection. Additionally, our findings imply that any interventions designed to improve knowledge of HBV infection, vaccination, and screening in the university should be sensitive to sociodemographic characteristics especially family history of HBV, ethnicity, and sex.

## Figures and Tables

**Table 1:** Sociodemographic characteristics of study participants.

Variable	Frequency (*n* = 386)	Percentage (%)
Age group		
<20	51	13.2
20–29	309	80.1
>29	26	6.7
Sex		
Male	147	38.1
Female	239	61.9
Religion		
Christian	315	81.6
Muslim	67	17.4
Traditionalist	4	1.0
Marital status		
Married	43	11.1
Single	342	88.6
Divorced	1	0.3
CWA range		
≥70	83	21.5
<70	303	78.5
Ethnicity		
Akan	254	65.8
Ewe	37	9.6
Ga-Adangbe	95	24.6
Programme		
Science-related courses	320	82.9
Business-related courses	66	17.1
Residence		
Rural	113	29.3
Urban	273	70.7
Occupation		
Employed	33	8.5
Unemployed	353	91.5

Data are presented as frequency (%). CWA: cumulative weighted average.

**Table 2:** Sociodemographic factors associated with knowledge on HBV infection among university students.

Variable	Total (*n* = 386)	Poor (*n* = 86)	Moderate (*n* = 101)	Adequate (*n* = 199)	*p* value
Sex					0.5060
Male	149 (38.60)	34 (39.53)	36 (35.64)	79 (39.70)	
Female	237 (61.40)	52 (60.47)	65 (64.36)	120 (60.30)	
Marital status					0.0850
Single	342 (88.60)	81 (94.19)	87 (25.44)	174 (87.44)	
Married	43 (11.14)	4 (4.65)	14 (13.86)	25 (12.56)	
Divorced	1 (0.26)	1 (1.16)	0 (0.00)	0 (0.00)	
Religion					0.3150
Christian	315 (81.61)	74 (86.05)	82 (82.00)	158 (79.40)	
Muslim	67 (17.36)	10 (11.63)	17 (17.00)	40 (20.10)	
Traditionalist	4 (1.04)	2 (2.33)	1 (1.00)	1 (0.50)	
Ethnicity					**0.0020**
Akan	254 (65.80)	68 (79.07)	72 (71.29)	114 (57.29)	
Ewe	95 (24.61)	11 (12.79)	19 (18.81)	65 (32.66)	
Ga-Adangbe	37 (9.59)	7 (8.14)	10 (9.90)	20 (10.05)	
Resident					0.7390
Rural	113 (29.27)	28 (32.56)	28 (27.72)	57 (28.64)	
Urban	273 (70.73)	58 (67.44)	73 (72.28)	142 (71.36)	
Occupation					
Employed	33 (8.55)	10 (11.63)	9 (8.91)	14 (7.04)	
Unemployed	353 (91.45)	76 (88.37)	92 (91.09)	185 (92.96)	
Family history of HBV infection					**0.0160**
Yes	48 (12.4)	3 (3.49)	16 (15.84)	29 (14.57)	
No	338 (87.6)	83 (96.51)	85 (84.16)	170 (85.43)	
CWA range					**0.0020**
≥70	83 (21.5)	30 (34.88)	16 (15.84)	37 (18.59)	
<70	303 (78.5)	56 (65.12)	85 (84.16)	162 (81.41)	
Program					0.6720
Business related	66 (17.1)	13 (15.12)	20 (19.80)	33 (16.58)	
Science related	320 (82.9)	73 (84.88)	81 (80.20)	166 (83.42)	

*Note*. Data are presented as frequency and percentage, *p* values are computed by the chi-square test, *p* value < 0.05 and bolded means statistically significant; CWA: cumulative weighted average of students' academic results, HBV: viral hepatitis B.

**Table 3:** Association between HBV testing, sex, and program of the study participants.

Variable	Total (*n* = 386)	HBV testing		*p* value	aOR (95% CI)	*p* value
		No (*n* = 187)	Yes (*n* = 199)			
Sex				0.0654^a^		
Male	147 (38.08)	80 (42.78)	67 (33.67)		1.00	—
Female	239 (61.92)	107 (57.22)	132 (66.33)		2.01 (0.78–3.98)	0.6020^b^
Program				<0.0001^**a**^		
Business related	66 (17.1)	47 (25.13)	19 (9.55)		1.00	—
Science related	320 (82.9)	140 (74.87)	180 (90.45)		2.56 (1.03–5.08)	0.0210^b^
Marital status				0.4956^a^		
Single	342 (88.60)	168 (89.8)	174 (87.4)		—	—
Married	43 (11.14)	20 (10.7)	25 (12.6)		—	—
Divorced	1 (0.26)	1 (0.5)	0 (0.0)		—	—
Religion				0.0649^a^		
Christian	315 (81.81)	144 (77.0)	171 (85.9)		—	—
Muslim	67 (17.36)	40 (21.4)	27 (13.6)		—	—
Traditionalist	4 (1.04)	3 (1.6)	1 (0.5)		—	—
Ethnicity				0.1761^a^		
Akan	254 (65.8)	113 (60.4)	141 (70.9)		—	—
Ewe	95 (24.61)	50 (26.7)	40 (20.1)		—	—
Ga-Adangbe	37 (9.59)	19 (10.2)	18 (9.0)		—	—
Resident				0.0573^a^		
Rural	113 (29.27)	46 (24.6)	67 (33.7)		—	—
Urban	273 (70.73)	141 (75.4)	132 (66.3)		—	—
Occupation				0.2018^a^		
Employed	33 (8.55)	12 (6.4)	21 (10.6)		—	—
Unemployed	353 (91.45)	175 (93.6)	178 (89.4)		—	—
Family history of HBV vaccination				0.0639^a^		
Yes	48 (12.4)	17 (9.1)	31 (15.6)		—	—
No	338 (87.6)	170 (90.9)	168 (84.4)		—	—
CWA range				0.1376^a^		
≥70	83 (21.5)	34 (18.2)	49 (24.6)		—	—
<70	303 (78.5)	153 (81.8)	150 (75.4)		—	—

Data are presented as frequency (%). ^a^*p* values computed using Chi-square or Fisher's exact test. aOR: adjusted odds ratio. ^b^*p* values computed using multivariate logistic regression adjusted for sex. *p* < 0.05 are considered statistically significant. *p* value < 0.05 and bolded means statistically significant.

**Table 4:** Association between awareness of HBV vaccination and sociodemographic characteristics of study participants.

Variable	Total (*n* = 386)	Low (*n* = 131)	Moderate (*n* = 128)	High (*n* = 127)	*p* value
Sex					**<0.0001**
Male	147 (38.08)	47 (35.88)	70 (54.69)	30 (23.62)	
Female	239 (61.92)	84 (64.12)	58 (45.31)	97 (76.38)	
Marital status					0.1280
Single	342 (88.60)	122 (93.13)	113 (88.28)	107 (84.25)	
Married	43 (11.14)	9 (6.87)	14 (10.94)	20 (15.75)	
Divorced	1 (0.26)	0 (0.00)	1 (0.78)	0 (0.00)	
Religion					0.3150
Christian	315 (81.81)	101 (77.10)	111 (86.72)	102 (80.95)	
Muslim	67 (17.36)	29 (22.14)	16 (12.50)	22 (17.46)	
Traditionalist	4 (1.04)	1 (0.76)	1 (0.78)	2 (1.59)	
Ethnicity					0.7020
Akan	254 (65.8)	88 (67.18)	83 (64.84)	83 (65.35)	
Ewe	95 (24.61)	30 (22.90)	30 (23.44)	35 (27.56)	
Ga-Adangbe	37 (9.59)	13 (9.92)	15 (11.72)	9 (7.09)	
Resident					0.2860
Rural	113 (29.27)	43 (32.82)	31 (24.22)	39 (30.71)	
Urban	273 (70.73)	88 (67.18)	97 (75.78)	88 (69.29)	
Occupation					0.1330
Employed	33 (8.55)	8 (6.11)	9 (27.3)	16 (48.5)	
Unemployed	353 (91.45)	123 (93.89)	119 (26.06)	111 (52.41)	
Family history of HBV vaccination					**0.0260**
Yes	48 (12.4)	8 (6.11)	20 (15.63)	20 (15.75)	
No	338 (87.6)	123 (93.89)	108 (84.38)	107 (84.25)	
CWA range					**0.0060**
≥70	83 (21.5)	16 (12.21)	34 (26.56)	33 (25.98)	
<70	303 (78.5)	115 (87.79)	94 (73.44)	94 (74.02)	
Program					**0.0020**
Business related	66 (17.1)	13 (9.92)	20 (15.63)	33 (25.98)	
Science related	320 (82.9)	118 (90.08)	108 (84.37)	94 (74.02)	

Data are presented as frequency and percentage, *p* values are computed by the chi-square test, *p* value < 0.05 and bolded means statistically significant; CWA: cumulative weighted average; HBV: hepatitis B virus.

## Data Availability

All data generated or analyzed during this study are included in this article and can be requested from the corresponding author.

## Abbreviations

HBV: Hepatitis B virus
CWA: Cumulative weighted average
GCUC: Garden City University College.
